# PANI-Coated VO_x_ Nanobelts with Core-Shell Architecture for Flexible All-Solid-State Supercapacitor

**DOI:** 10.3390/mi14101856

**Published:** 2023-09-28

**Authors:** Qiang Zhang, Xianran Li, Yinyin Zheng, Qian Tu, Shiwen Wei, Hong Shi, Wentao Tang, Liangzhe Chen

**Affiliations:** School of Electronic Information Engineering, Jingchu University of Technology, Jingmen 448000, China; zq10183110@126.com (Q.Z.); ggll925597@163.com (X.L.); z13299251453yy@163.com (Y.Z.); tq010406@126.com (Q.T.); wsw_1205@163.com (S.W.); shirley1441@163.com (H.S.)

**Keywords:** VO_x_ nanobelts, PANI, core-shell architecture, flexible, supercapacitor, electronics

## Abstract

As a typical pseudocapacitor material, VO_x_ possesses mixed valence states, making it an ideal electrode material for symmetric screen-printed supercapacitors. However, its high internal resistance and low energy density are the main hurdles to its widespread application. In this study, a two-dimensional PANI@VO_x_ nanobelt with a core-shell architecture was constructed via a two-step route. This strategy involves the preparation of VO_x_ using a solvothermal method, and a subsequent in situ polymerization process of the PANI. By virtue of the synergistic effect between the VO_x_ core and the PANI shell, the optimal VO_x_@PANI has an enhanced conductivity of 0.7 ± 0.04 S/Ω, which can deliver a high specific capacitance of 347.5 F/g at 0.5 A/g, a decent cycling life of ~72.0%, and an outstanding Coulomb efficiency of ~100% after 5000 cycles at 5 A/g. Moreover, a flexible all-solid-state symmetric supercapacitor (VO_x_@PANI SSC) with an in-planar interdigitated structure was screen-printed and assembled on a nickel current collector; it yielded a remarkable areal energy density of 115.17 μWh/cm^2^ at an areal power density of 0.39 mW/cm^2^, and possessed outstanding flexibility and mechanical performance. Notably, a “Xiaomi” hygrothermograph (3.0 V) was powered easily by tandem SSCs with an operating voltage of 3.1 V. Therefore, this advanced pseudocapacitor material with core-shell architecture opens novel ideas for flexible symmetric supercapacitors in powering portable/wearable products.

## 1. Introduction

With the tremendous development of portable/wearable products, the pursuit of advanced electronic energy storage (EES) devices has been stimulated with the merits of flexibility, durability, ease of processability, and environmental friendliness [1,2,3,4,5]. Of the various optional flexible EES devices, supercapacitors (also well-known as electrochemical capacitors) with their in-planar interdigitated structures are extensively perceived as promising EES devices due to their short charge times, high power densities, and long cycle lives, which have the potential to miniaturize electronics [6,7,8]. However, most supercapacitors suffer from cumbersome and high-cost fabrication methods, e.g., electrodeposition [9], laser etching [10], thermal evaporation [11], and sputtering [12]. As an attractive printing technique, screen printing, by virtue of its high throughput, good compatibility, and low cost [13,14], demonstrates great potential for supercapacitor manufacturers, who can transfer the ink directly from a stencil to the surfaces of various substrates, so that the roll-to-roll method can be abandoned [15,16,17]. Therefore, the main challenge that must be faced is the development of high-performance electrode materials. 

Currently, the matching of positive and negative materials is still an obstruction, and the primary sticking point is the diversity in specific capacitances for the anode and cathode [18,19,20,21]. Additionally, different electrode materials mean that they need to be printed twice or more, which imposes extra burdens for operations and costs. Hence, an outstanding material is urgently needed that can be applied synchronously in the anode and cathode. More recently, much attention has been paid to the development of transition metal oxides, i.e., Co_3_O_4_ [22], MnO_2_ [23], VO_2_ [24], etc. As a typical pseudocapacitor material, vanadium oxide, especially for VO_x_, possesses mixed valence states, and is deemed an ideal electrode material for supercapacitors owing to its high theoretical specific capacitance [25,26,27]. Nevertheless, its high internal resistance and low energy density on account of its poor conductivity are the main hurdles to its widespread application [28]. Taking full advantage of its high conductivity and ease of preparation, conductive polymers provide an opportunity to overcome the above issues, while PANI coating has been verified to be an effective means in current research [29,30,31]. Therefore, there is an enormous potential for PANI coatings on the surfaces of VO_x_ nanobelts for constructing high-performance electrode materials with core-shell nanostructures. 

In this study, we report a two-step approach to prepare PANI@VO_x_ nanobelts with a core-shell architecture. Initially, a two-dimensional VO_x_ nanobelt was synthesized via a facile solvothermal method, and an ultrathin coating layer (~35.7 nm) of PANI (as a shell) on the surface of the VO_x_ nanobelt (as a core) was obtained after in situ polymerization. Within the VO_x_@PANI nanobelt, the rough surface of the PANI not only can offer a larger specific surface area and a more active site for the electrochemical reaction, but it also has a relatively outstanding conductivity that enables the fast migration of electrons during the charge/discharge process. Additionally, making full use of the significant synergistic effect between the VO_x_ core and PANI shell, the VO_x_@PANI electrodes show superior electrochemical performance compared to pristine VO_x_ electrodes, including a higher specific capacitance, a longer cycling life, and a lower charge transfer resistance. Moreover, a flexible all-solid-state symmetric supercapacitor (VO_x_@PANI SSC) with an in-planar interdigitated structure was screen-printed and assembled on a nickel current collector, and achieved outstanding flexibility and mechanical properties as well as a remarkable energy density. Furthermore, a “Xiaomi” hygrothermograph (3.0 V) was easily powered by tandem SSCs, indicating the vast potential of the supercapacitor for energy storage applications. 

## 2. Experimental

### 2.1. Materials

The vanadium pentaoxide (V_2_O_5_), ammonium persulfate (APS), absolute ethanol (EtOH), sodium sulfate (Na_2_SO_4_), N-methyl-2-pyrrolidone (NMP), and polyvinyl alcohol (PVA) were purchased from Shanghai Maclin Biochemical Technology Co., Ltd., Shanghai, China. Aniline (ANI, ≥99.5%) was traded from the Shanghai Aladdin Biochemical Technology Co., Ltd., Shanghai, China. The nickel foam (1.0 mm in thickness) and polyvinylidene fluoride emulsion (PVDF, 3.0 wt%) were purchased from Saibo Electrochemical Materials Co., Ltd., Shenzhen, China. The polyethene terephthalate (PET, 0.2 mm in thickness) film was traded commercially. All of the reagents were of analytical purity without being treated further. Deionized water (H_2_O) was used throughout the whole experiment.

### 2.2. Synthesis of VO_x_ Nanobelt

The VO_x_ nanobelt was synthesized via a facile solvothermal method. Typically, 5.0 mmol of V_2_O_5_ powder was dissolved in 40.0 mL of EtOH/H_2_O solution (the volume ratio was 1:3), and was transferred into a Teflon-lined stainless steel autoclave. Then, the autoclave was sealed and maintained at 180 °C for 12 h. After cooling to room temperature, the residue was collected via centrifugation and washed with EtOH several times. Finally, the black-grey VO_x_ was obtained by drying in a vacuum at 80 °C for 12 h.

### 2.3. Synthesis of VO_x_@PANI Core-Shell Nanobelt

The PANI shell was deposited onto the surface of the VO_x_ nanobelt through the in situ polymerization of aniline. Initially, 0.5 g of VO_x_ was added into 35.0 mL of HCl solution (0.1 mol/L), followed by the dropwise addition of redistilled aniline under continuous stirring. Then, 1.17 g of APS was dissolved in 15.0 mL of HCl solution (0.1 mol/L), followed by its dropwise addition into the mixture that was kept at 3 °C for 2 h. Lastly, the product was collected by washing and drying in a vacuum at 80 °C. All of the experiments were conducted in an ice-water bath. According to the additional volumes of aniline with 0.16 mL, 0.48 mL, and 0.80 mL, the results were labelled as V@P-1, V@P-3, and V@P-5, respectively. 

For comparison, an experiment without the addition of VO_x_ was conducted to obtain the PANI product.

### 2.4. Fabrication of Symmetric VO_x_@PANI Supercapacitor

Typically, the procedure for manufacturing the supercapacitors can be summarized in three steps. In step one, 80 wt% VO_x_@PANI, 10 wt% active carbon, and 10 wt% PVDF were mixed to form a homogeneous slurry, and NMP was employed to modulate the viscosity as required. In step two, the prepared ink was screen-printed on the Ni foam, together with drying in a vacuum for 80 °C. In step three, the prepared PVA/Na_2_SO_4_ gel (4.0 g of PVA and 1.4 g of Na_2_SO_4_ were dissolved in 40 mL of H_2_O, with continuous stirring at 90 °C until it became a clear and transparent gel, and then the mixture was rested in the air at 25 °C overnight) was evenly covered over it, and the flexible all-solid-state VO_x_@PANI was obtained after naturally drying in the air overnight.

### 2.5. Electrochemical Testing

The electrochemical tests, including cyclic voltammetry (CV), galvanostatic charge and discharge (GCD), electrochemical impedance spectroscopy (EIS), cycling stability, etc., were performed on a CorrTest electrochemical workstation (CS350H, CorrTest Instruments Co., LTD, Wuhan, China). For the electrodes, the prepared ink (80 wt% of the composite, 10 wt% active carbon, and 10 wt% PVDF were mixed to form a homogeneous slurry) was coated onto a nickel foam of dimensions 1.0 cm × 2.0 cm and dried in a vacuum at 80 °C overnight. A three-electrode system, composed of a working electrode, an Ag/AgCl reference electrode, and a Pt plate counter electrode, was used to evaluate the electrochemical performances of the electrodes. In the three-electrode system, the working electrode was dipped ~1.0 cm into the Na_2_SO_4_ electrolyte (1.0 mol/L) at room temperature. The mass-specific capacitance (*C*, F/g) could be counted according to the galvanostatic charge/discharge (GCD) curves based on the following equation [32,33]:

(1) $$C=\frac{I\times ∆t}{m\times ∆V}$$

where *I*, ∆*t*, *m*, and ∆*V* are the current density, the discharge time, the mass loading of active materials, and potential windows, respectively. As for the supercapacitors, the electrochemical performance was assessed in a two-electrode system, in which the VO_x_@PANI electrode acts as both the positive and negative electrodes to fabricate the symmetric supercapacitor.

### 2.6. Characterization

The morphology and elemental mapping were investigated using a scanning electron microscope (SEM, Zeiss SIGMA, Darmstadt, Germany) under an accelerating voltage of 15.0 kV with an energy dispersive spectrometer (EDS, Oxford X-MAX, Oxford, UK). The composition and valence bonds were characterized via X-ray photoelectron spectroscopy (XPS, ThermoFisher EscaLab250Xi, Waltham, MA, USA) using Al-Kα radiation. The specific surface area and the pore diameter distribution were measured on a specific surface area analyzer (Micromeritics ASAP 2460, Norcross, GA, USA) at 77 K. The crystalline structure was measured on an X-ray diffractometer (XRD, Rigaku Mini Flex600, Tokyo, Japan) with Cu-Kα radiation (λ = 1.5406 Å).

## 3. Results and Discussion

### 3.1. Characterization

**Figure 1:**
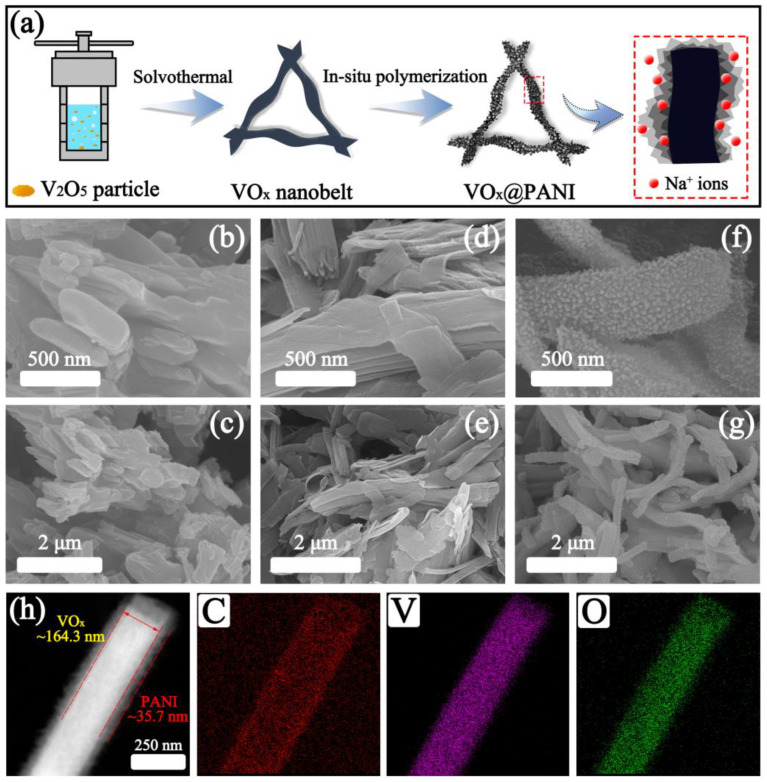
(**a**) Schematic diagram of the preparation of the VO_x_@PANI nanobelts, SEM images of (**b**,**c**) V_2_O_5_, (**d**,**e**) VO_x_ nanobelts, (**f**,**g**) VO_x_@PANI, and (**h**) EDS mapping of V@P-3 composite with C, V, and O elements.

The growth mechanism and formation process of the VO_x_@PANI nanobelts using a facile two-step route is illustrated in Figure 1a. In the beginning, V_2_O_5_ nanoparticles with an irregular shape and different sizes (Figure 1b,c) are translated to hierarchical VO_x_ nanobelts via a solvothermal method at 180 °C (Figure 1d,e). It is worth noting that the average length and width of the nanobelts were calculated to be approximately 1.69 μm and 0.31 μm, respectively, and the length/width ratio accounted for ~5.5, according to the SEM image (Figure S1). Then, the aniline monomers were adsorbed to the surface of the VO_x_ nanobelts and oxidized by ammonium persulfate under an ice bath. After that, the well-designed VO_x_@PANI nanobelts with a core-shell construction were collected (Figure 1f,g). In addition, it was found that the additional amount of aniline had an enormous impact on the morphology of the VO_x_@PANI nanobelt, as shown in Figure S2. In the V@P-1 sample, the PANI shell is thin, while several grains are presented on the surface (Figure S2a,b). With an increase in aniline, these grains grow in volume and quantity (Figure 1f,g), which can be ascribed to the oxidation of excess aniline to polyaniline. However, this special texture fades away in the V@P-5 sample, while bare VO_x_ nanobelts and PANI nano-branches occur instead (Figure S2c,d). The reason may be that most of the APS oxidants depleted the superfluous aniline, inducing the in situ polymerization and formation of PANI nano-branches. More importantly, it should be emphasized that a rougher surface can provide a larger specific surface area, tremendously increase the contact area for the electrolytes, and boost ion (Na^+^) diffusion and transport in the electrochemical process [34].

To verify this claim, the specific surface area analysis was adopted, including the N_2_ adsorption/desorption isotherms and the BJH pore size distribution curves, as exhibited in Figure S3a. The typical type-IV adsorption with an H3 hysteresis loop suggests an abundant mesoporous structure in the two samples [35], while the average BJH pore diameter of the VO_x_ and V@P-3 are 6.16 nm and 7.14 nm, respectively, as shown in Figure S3b. Additionally, owing to the rough PANI shell, the V@P-3 composite possesses a contented specific surface area of 108.3 m^2^/g with a pore volume of 0.19 cm^3^/g, which is larger than that of the VO_x_ (85.2 m^2^/g and 0.17 cm^3^/g, respectively). Figure 1h indicates the elemental distribution of the V@P-3 composite, in which the C, V, and O elements overlap well, and the molar ratio of the C and V elements was calculated as ~3.8 based on the elemental content in Table S1. Furthermore, according to the EDS mapping images, the thickness of the PANI shell is approximately ~35.7 nm, while the width of the VO_x_ core is measured as ~164.3 nm.

**Figure 2:**
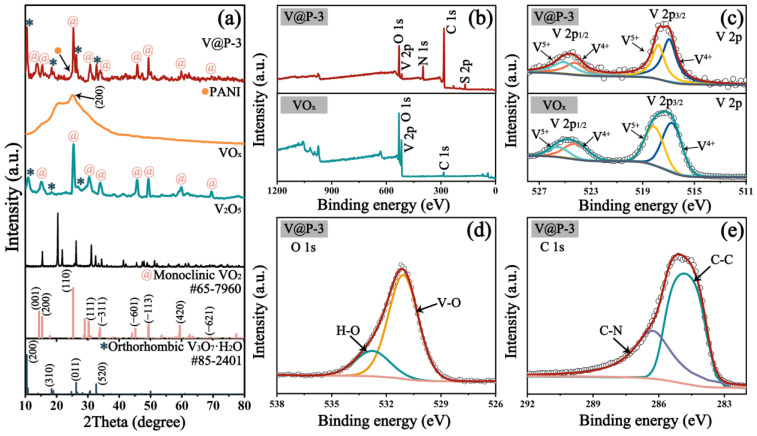
(**a**) XRD patterns of various samples; (**b**) XPS survey spectrum and core spectrum of (**c**) V 2p, (**d**) C 1s, and (**e**) O 1s of VO_x_ and V@P-3 composites.

The crystal texture of the V_2_O_5_, VO_x_, PANI, and V@P samples were characterized using XRD, and the results are shown in Figure 2a and Figure S4. The VO_x_ pattern is composed of a dominant monoclinic VO_2_ crystal phase (JCPDS #65-7960) [36] and a minor orthorhombic V_3_O_7_·H_2_O crystal phase (JCPDS #85-2401) [37]. Moreover, the diffraction peaks located at 2θ = 15.2°, 25.4°, 30.4°, 34.0°, 45.8°, 49.3°, 59.9°, and 69.4° correspond to the (200), (110), (111), (–311), (–601), (–113), (420), and (–621) planes of VO_2_ (marked by the symbol @) [38], while those of the peaks centered at 2θ = 15.2°, 18.3°, and 26.8° belong to the (200), (310), and (011) planes of V_3_O_7_·H_2_O (marked by the symbol ∗) [39]. After coating with PANI (marked by the symbol *●*), a wide peak pack derived from the characteristic peak of the (002) lattice plane comes out at approximately 2θ = 25° [40], confirming the successful preparation of the VO_x_@PANI composite. Interestingly, the diffraction peaks attributed to the (001) lattice plane (2θ = 13.8°) of the VO_2_ and the (520) crystal face (2θ = 32.7°) of the V_3_O_7_·H_2_O were detected in the VO_x_@PANI samples, which may be due to the improvement in crystallinity for the VO_x_ composites. XPS analyses were employed to further investigate the chemical states of the V, O, N, C, and S elements within the VO_x_@PANI composite, while only the V, O, and C elements exist in the VO_x_ sample (Figure 2b). The V 2p spectrum of the VO_x_ (Figure 2c) exhibits two major peaks of V 2p_1/2_ and V 2p_3/2_ spin orbits, which can be further revolved into four peaks. Among them, the peaks at 516.7 and 524.1 eV are assigned to V^4+^, while those at 518.1 and 525.5 eV correspond to V^5+^ [41,42], confirming the coexistence of the VO_2_ and V_3_O_7_·H_2_O composites. After coating with the PANI layer, the valences of the V species are consistent except for several shifts in the binding energies for V^4+^ (516.9 and 524.2 eV) and V^5+^ (517.8 and 525.1 eV). Figure 2d demonstrates the O 1s spectrum with two peaks at 531.1 and 532.8 eV, indexing to the V-O and H-O bonds, respectively [43]. The C 1s spectrum in Figure 2e is deconvoluted into three peaks, where the peaks at 284.8 and 286.3 eV are related to the C-C and C-N bonds that originated from the PANI coating on the VO_x_ [44,45].

### 3.2. Electrochemical Performances

**Figure 3:**
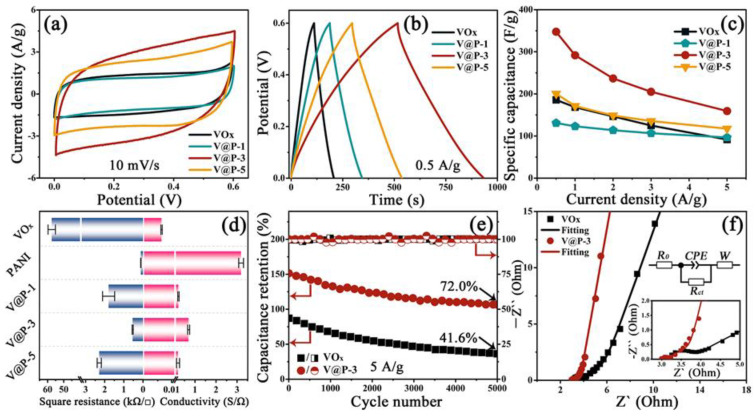
(**a**) CV curves at 10 mV/s, (**b**) GCD curves under 0.5 A/g, (**c**) specific capacitances at various current densities, and (**d**) the square resistances and electric conductivities of VO_x_, V@P−1, V@P−3, and V@P−5 composites; (**e**) cycling stability and Coulombic efficiency under 5 A/g for 5000 charge/discharge cycles and (**f**) the Nyquist plots of VO_x_ and V@P−3 electrodes; the inset shows the equivalent circuit and the Nyquist plots at high-frequency region.

Aiming to explore the merits of the core-shell architecture for the VO_x_@PANI composite, the electrochemical performances are measured on a three-electrode system. Figure 3a presents the CV curves of the VO_x_, V@P-1, V@P-3, and V@P-5 electrodes at a scan rate of 10 mV/s, in a potential window from 0 to 0.6 V. A nearly rectangular shape without distinct redox peaks suggests the typical pseudocapacitance characteristics for all of the samples [46]. Compared with the other samples, the V@P-3 electrode owns the largest integral area of the CV curve, indicating a superior capacitive performance. When the scan rate increases from 10 to 50 mV/s, the shape of the CV curve is well-maintained (Figure S5), revealing the outstanding rate capability. According to previous research, the charge/discharge process of the VO_x_@PANI composite is mainly dominated by a diffusion-controlled intercalation pseudocapacitance coupled with a minor surface-controlled redox pseudocapacitance [46]. The possible electrochemical reaction in the electrolytes can be described as follows [47]:

(2) $${VO}_{x}+\mathrm{yNa}++\mathrm{ye}-↔\mathrm{Na}y\mathrm{VO}x$$

Figure 3b compares the GCD profiles of the VO_x_, V@P-1, V@P-3, and V@P-5 electrodes at a current density of 0.5 A/g, among which the V@P-3 electrode possesses the longest discharge time, as identified in the CV results. In addition, the quasi-symmetric part in the charge and discharge times clearly shows the good electrochemical reversibility of all of the samples. The corresponding specific capacitances are acquired from the GCD curves (Figure S6) according to Equation (1), and the calculations are listed in Figure 3c. When the current density is 0.5 A/g, the specific capacitances of the VO_x_, V@P-1, V@P-3, and V@P-5 electrodes are calculated to be 185.8 F/g, 267.1 F/g, 347.5 F/g, and 200.5 F/g, respectively. Note that the specific capacitance of the V@P composites is higher than that of the VO_x_ electrode; this may come down to the significant synergistic effect between VO_x_ with its high pseudocapacitance as a core and the highly conductive PANI as a shell. On the one side, the rough surface of the PANI can offer a larger specific surface area and a more active site for electrochemical reactions. On the other side, the PANI has a relatively outstanding conductivity, and enables the rapid migration of electrons in the charge/discharge process. The results of the four-probe square resistance tester in Figure 3d verified this assumption. Distinctly, the PANI sample has the lowest square resistance (0.1 ± 0.008 kΩ/□) and the greatest conductivity (3.2 ± 0.1 S/Ω), while the VO_x_ sample has the largest square resistance (57.5 ± 2.2 kΩ/□) and the poorest conductivity (0.006 ± 0.0002 S/Ω). After coating with PANI on the surface of the VO_x_ nanobelts, the electrical conductivity increased prodigiously. Among the results, the conductivity of the V@P-3 sample (0.7 ± 0.04 S/Ω) is much better than those of the V@P-1 (0.2 ± 0.03 S/Ω) and V@P-5 (0.2 ± 0.07 S/Ω) samples; this is probably due to the optimal core-shell construction for the V@P-3 sample, which can account for the largest specific capacitance for the V@P-3 electrode. Accordingly, the specific capacitance of the V@P-3 electrode always takes the leading position, even at a high current density.

Additionally, the V@P-3 electrode shows a satisfactory stability property after 5000 charge/discharge cycles at 5 A/g, as ~72.0% of the initial capacitance, while only approximately 41.6% is retained on the VO_x_ electrode (Figure 3e). Note that the high Coulombic efficiency of the two electrodes (around 100%) indicates an equal charge and discharge time, suggesting that the decline in capacitance primarily results from diffusion. Furthermore, the impedances of the VO_x_ and V@P-3 electrodes were obtained from the electrochemical impedance spectroscopy (EIS) analysis before cycling. The Nyquist plots of the two electrodes in Figure 3f comprise a semicircle at a high-frequency region and a straight line at a low-frequency region. The equivalent circuit diagram can be found in the illustration of Figure 3f, wherein the parameters of *R*_0_, *CPE*, *R_ct_*, and *W* represent the electrolyte resistance, charge transfer resistance, constant phase angle content, and Warburg resistance, respectively [33], and the fitting results are listed in Table S2. As expected, the V@P-3 electrode delivers lower *R*_0_ (3.01 Ω) and *R_ct_* (0.12 Ω) values in comparison with those of the VO_x_ electrode (*R*_0_ = 3.16 Ω, *R_ct_* = 0.48 Ω), elucidating superior electrical conductivity and faster charge transfer for the V@P-3 composite.

Based on the above analysis, the mechanism of the excellent capacitive and stability performances of VO_x_@PANI-3 are explained as follows. As a highly conductive polymer, the PANI shell with a rough and porous surface provides more sites for electrochemical reactions, as diagramed in Figure 1a. On the one hand, the encapsulated PANI can boost the migration of sodium ions and hasten charge transfer, generating a superior specific capacitance. On the other hand, this shell is able to confine the excessive dissolution of VO_x_ nanoparticles in the continuous charge/discharge process, inducing a long cycling life [48]. 

**Figure 4:**
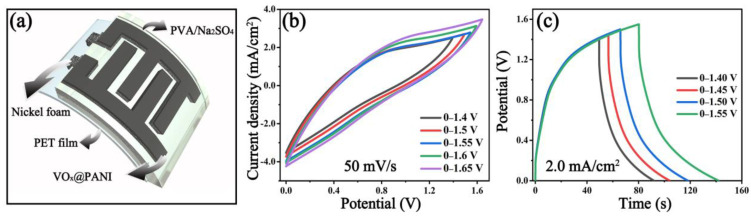
(**a**) Structural diagram of the flexible VO_x_@PANI supercapacitor, (**b**) CV curves at 50 mV/s, and (**c**) GCD curves at 2.0 mA/cm^2^ of VO_x_@PANI SSC under different potential windows.

To highlight the VO_x_@PANI electrode material in the energy storage device, a flexible supercapacitor that consists of the VO_x_@PANI as positive and negative active materials, PVA/Na_2_SO_4_ as the electrolyte, and Ni foam as a current collector was assembled on a PET substrate (Figure 4a). Figure 4b shows the CV curves of the VO_x_@PANI SSC at a scan rate of 50 mV/s in different potential windows ranging from 1.40 to 1.65 V. Since there is good consistency in the CV curves, the operating voltage can be extended to 1.55 V after taking full advantage of the pseudocapacitive VO_x_@PANI. The GCD profiles in Figure 4c at a current density of 2.0 mA/cm^2^ in potential windows from 1.40 to 1.55 V support this viewpoint, in which the GCD curves in the charge part overlap well.

**Figure 5:**
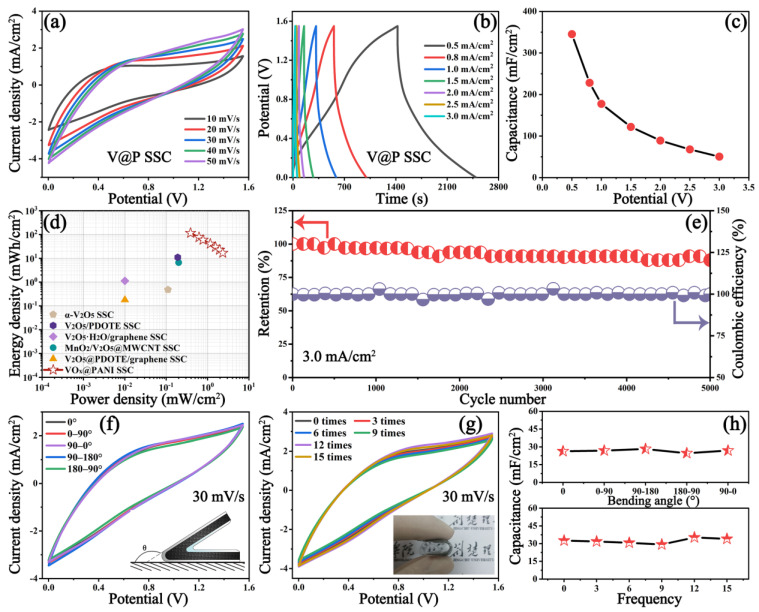
(**a**) CV curves of VO_x_@PANI SSC at different scan rates, (**b**) GCD curves at different current densities, (**c**) the corresponding specific capacitance, (**d**) the Ragone plots, (**e**) the cycling stability and Coulombic efficiency at 3.0 mA/cm^2^. CV curves acquired at 30 mV/s at different (**f**) bending angles (illustration shows the beding schematic diragram) and (**g**) bending frequencies (illustration exhibits the beding photograph), and (**h**) the corresponding areal specific capacitances.

The detailed electrochemical performances of VO_x_@PANI SSC are summarized in Figure 5. All of the CV curves with a potential of 1.55 V at various scan rates in Figure 5a show a similar shape, suggesting a good rate capability. The capacitive properties were further investigated via the approximately triangular GCD curves (Figure 5b) at current densities ranging from 0.5 to 3.0 mA/cm^2^, and the areal specific capacitance (*C_s_*, mF/cm^2^) was calculated according to the following equation [49]:

(3) $$C_{s}=\frac{I\times ∆t}{S\times ∆V}$$

where *S* is the effective area of the screen-printed SSC. A remarkable *C_s_* of 345.2 mF/cm^2^ can be seen in Figure 5c at 0.5 mA/cm^2^, which still remains 50.3 mF/cm^2^ at a high current density of 5.0 mA/cm^2^. Additionally, the areal energy density (*E*, mWh/cm^2^) and the power density (*P*, mW/cm^2^) of the VO_x_@PANI SSC were obtained using the following equations [50]:

(4) $$E=\frac{1}{2}C_{s}\times ∆V^{2}$$

(5) $$P=\frac{E}{∆t}$$

where ∆*V* is the potential window and ∆*t* is the discharge time.

Following calculations, the results are summarized in the Ragone plots (Figure 5d). Significantly, the VO_x_@PANI SSC can yield an ultrahigh areal energy density of 115.17 μWh/cm^2^ at an areal power density of 0.39 mW/cm^2^, while achieving an impressive areal power density of 2.33 mW/cm^2^ at an areal energy density of 16.8 μWh/cm^2^. These values are substantially superior to the reported vanadium oxide-based supercapacitors, such as α-V_2_O_5_ SSC (0.48 µWh/cm^2^ at 0.11 mW/cm^2^) [51], V_2_O_5_/PDOTE SSC (11.00 µWh/cm^2^ at 0.19 mW/cm^2^) [52], V_2_O_5_@PDOTE/graphene SSC (0.18 µWh/cm^2^ at 0.01 mW/cm^2^) [53], V_2_O_5_·H_2_O/graphene SSC (1.13 µWh/cm^2^ at 0.01 mW/cm^2^) [54], and MnO_2_/V_2_O_5_@MWCNT SSC (6.58 µWh/cm^2^ at 0.20 mW/cm^2^) [55] (Table S2). Moreover, after 5000 charge and discharge cycles at 3.0 mA/cm^2^, a satisfactory capacitance retention of ~87.9% and Coulombic efficiency of ~99.2% are still retained on the VO_x_@PANI SSC, showing its prodigious application prospects in electronics (Figure 5e). Furthermore, the flexibility and durability of the SSC were assessed by bending it at different angles or folding it several times, and the corresponding CV curves at 30 mV/s are shown in Figure 5f,g. It is also impressive that the CV curves overlapped well, even after bending the SSC 180° or folding it 15 times. The areal-specific capacitances (*C_s_*, mF/cm^2^) from the CV profiles were calculated as follows:

(6) $$C_{S}=\frac{\int I(V)dV}{S\times v\times ∆V}$$

where *∫I(V)dV* and *v* are the enclosed areas of the CV curve and the scan rate, respectively. As shown in Figure 5h, the *C_s_* value fluctuates slightly from 24.6 to 28.2 mF/cm^2^ after bending the sample at different angles, while varying from 29.2 to 35.2 mF/cm^2^ after folding the device several times, proving its prominent flexibility and durability, and making it applicable for flexible electronic products. 

**Figure 6:**
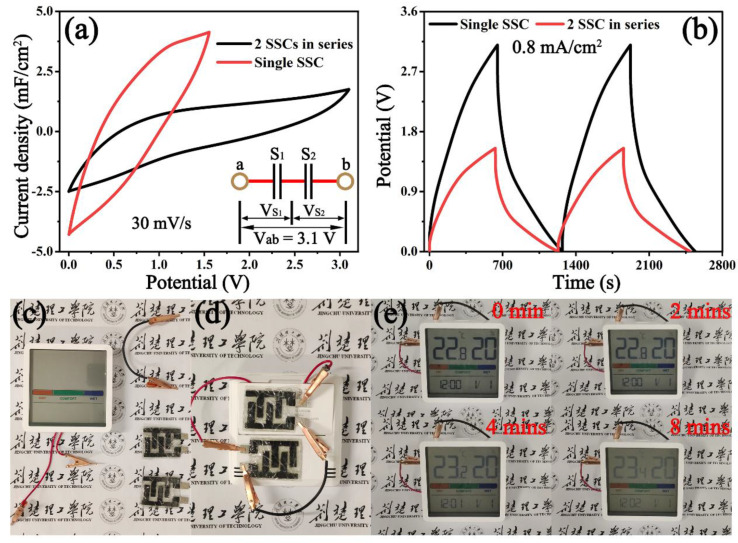
(**a**) CV curves at 30 mV/s and (**b**) GCD curves at 1.0 mA/cm^2^ of the single SSC and two SSCs in series, (**c**) optical images of the hygrothermograph, wires, and SSCs, (**d**) two SSCs connected in series, and (**e**) a hygrothermograph (3.0 V) powered for 8 min.

To further verify the practical applications, two SSC devices were connected in series. As depicted in the CV curve at 30 mV/s (Figure 6a) and the GCD curve at 0.8 mA/cm^2^ (Figure 6b), the potential window of the tandem SSCs (3.1 V) increased to double its original value (1.55 V). As a demonstration in Figure 6c–e, tandem SSCs can drive a “Xiaomi” hygrothermograph (3.0 V) with ease, and keep it working for more than 8 min; this result further confirms the vast application potential in energy conversion.

## 4. Conclusions

In summary, we proposed a two-step approach that combines a solvothermal method and an in situ polymerization mode to prepare PANI@VO_x_ nanobelts with core-shell architectures, in which there is an ultrathin coating layer (~35.7 nm) of PANI shell on the surface of the VO_x_ nanobelt core. By making full use of the significant synergistic effect, the optimal VO_x_@PANI has a superior conductivity of 0.7 ± 0.04 S/Ω, which can deliver an excellent specific capacitance of 347.5 F/g at 0.5 A/g, a decent cycling life of ~72.0%, and an outstanding Coulomb efficiency of ~100% after 5000 cycles at 5 A/g. Moreover, the assembled all-solid-state VO_x_@PANI SSC achieves a maximum areal energy density of 115.17 μWh/cm^2^ at an areal power density of 0.39 mW/cm^2^, as well as possessing outstanding flexibility and mechanical performance. Notably, a “Xiaomi” hygrothermograph (3.0 V) powered by two SSCs connected in series was sustained for more than 8 min. Furthermore, the approach used in this research can be extended to the construction of pseudocapacitor materials with core-shell architectures, which opens new ideas for flexible symmetric supercapacitors in powering portable/wearable products.

## Data Availability

Not applicable.

## Supplementary Materials

The following supporting information can be downloaded at: https://www.mdpi.com/article/10.3390/mi14101856/s1. Figure S1 SEM images of (a) V@P-3 hybrid; the corresponding statistical histogram of (b) length and (c) width. Figure S2 SEM images of (a,b) V@P-1 and (c,d) V@P-5. Figure S3 (a) N2 adsorption/desorption isotherms and (b) the BJH pore size distribution curves of VOx and V@P-3 samples. Figure S4 XRD patterns of V@P-1, V@P-3 and V@P-5. Figure S5 CV curves of V@P-3 electrode at different scan rates ranging from 10 to 50 mV/s. Figure S6 GCD curves at different current densities ranging from 0.5 to 5 A/g of (a) VOx, (b) V@P-1, (b) V@P-3, and (b) V@P-5 electrode. Table S1 The content of C, N, O, V elements for V@P-3 composite by EDS analysis. Table S2 Impedance values of the VO_x_ and V@P-3 according to the equivalent circuit. Table S3 Comparison of areal power density and energy density for VO_x_@PANI SSC and other reported vanadium oxide-based supercapacitors.
