# Supplementary material for: PANI-Coated VOx Nanobelts with Core-Shell Architecture for Flexible All-Solid-State Supercapacitor
Source: Micromachines (Basel). 2023 Sep 28;14(10):1856. doi: 10.3390/mi14101856 (PMC10609290; doi:10.3390/mi14101856)
Supplement: Supplementary file 1 [file micromachines-14-01856-s001.zip › micromachines-2624509-supplementary.pdf]

Supporting Information

PANI-coated VO_x nanobelts with core-shell architecture for flexible all-solid-state supercapacitor

Qiang Zhang, Xianran Li, Yinyin Zheng, Qian Tu, Shiwen Wei, Hong Shi, Wentao Tang*, Liangzhe Chen*

School of Electronic Information Engineering, Jingchu University of Technology, Jingmen 448000, China

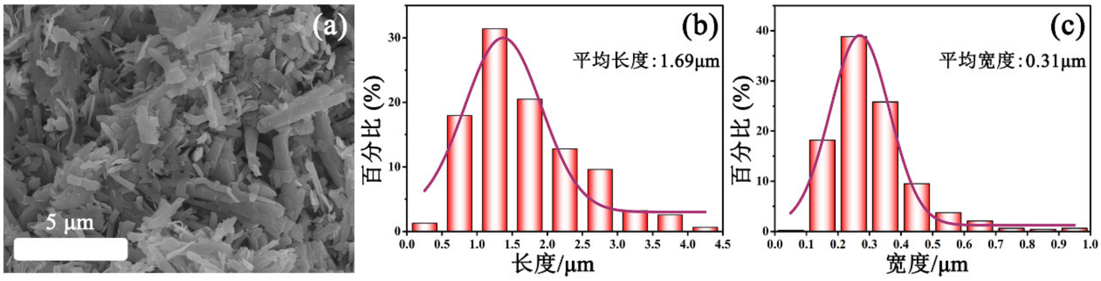

Figure S1 SEM images of (a) V@P-3 hybrid; the corresponding statistical histogram of (b) length and (c) width.

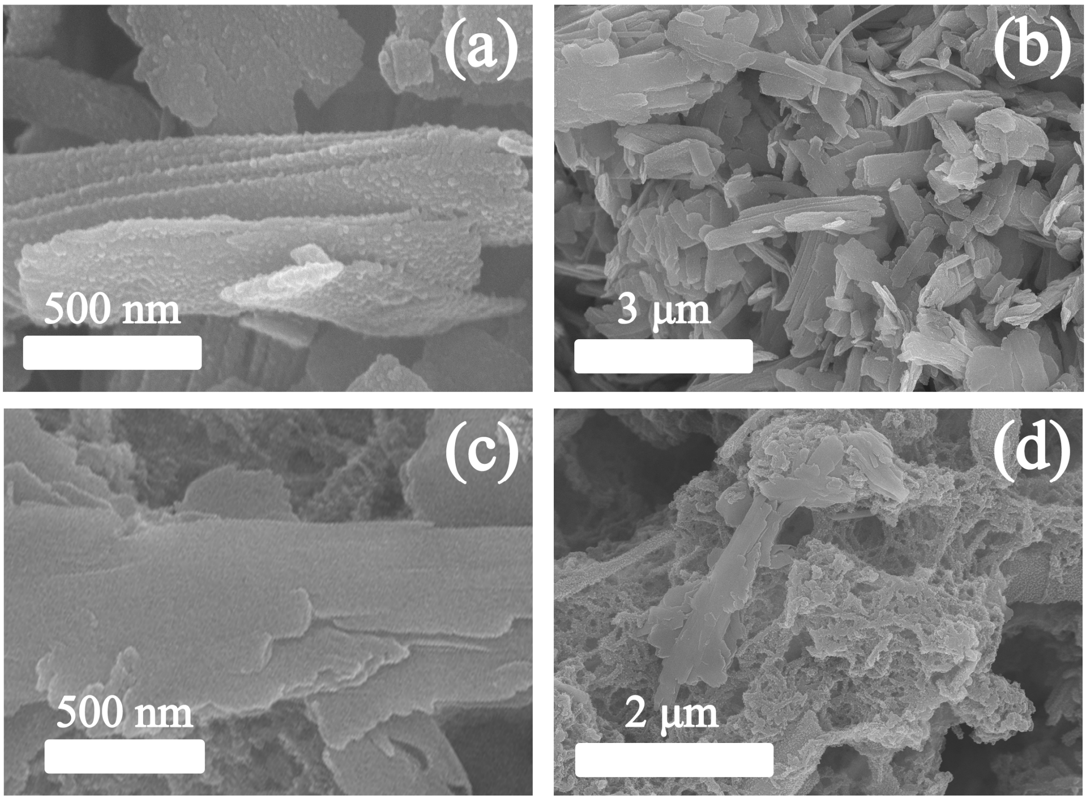

Figure S2 SEM images of (a, b) V@P-1 and (c, d) V@P-5.

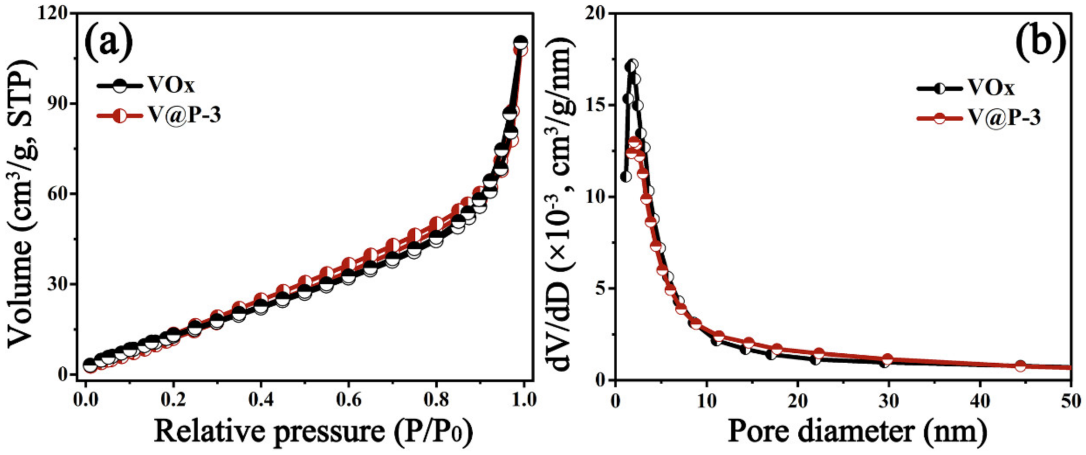

Figure S3 (a) N_2 adsorption/desorption isotherms and (b) the BJH pore size distribution curves of VO_x and V@P-3 samples.

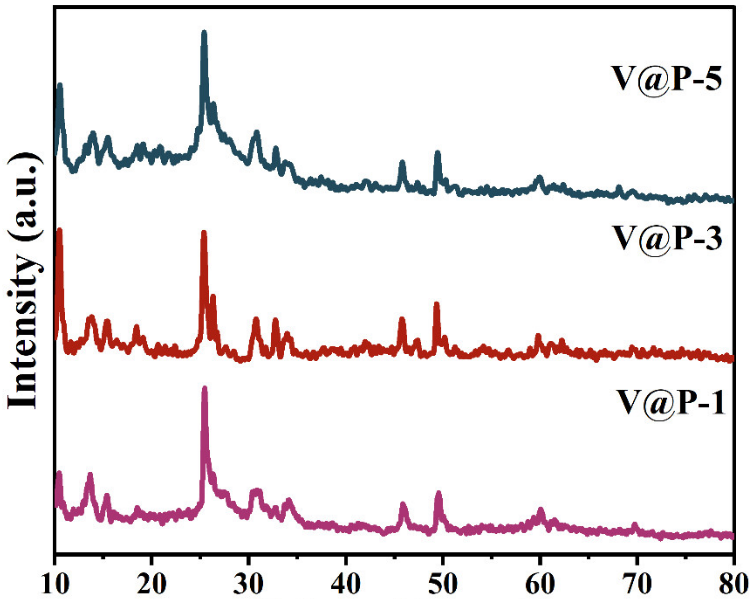

Figure S4 XRD patterns of V@P-1, V@P-3 and V@P-5.

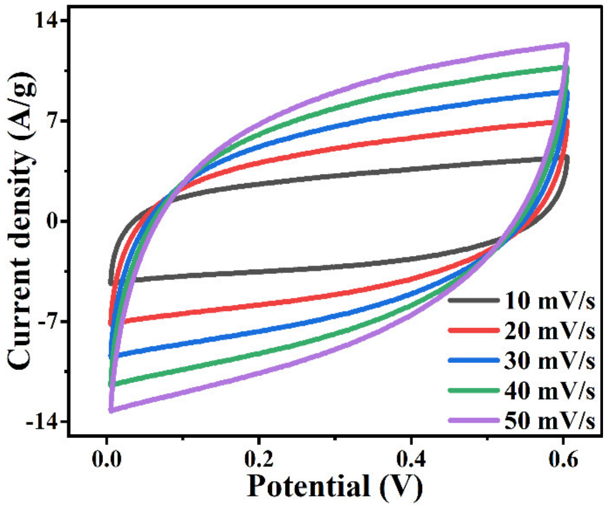

Figure S5 CV curves of V@P-3 electrode at different scan rates ranging from 10 to 50 mV/s.

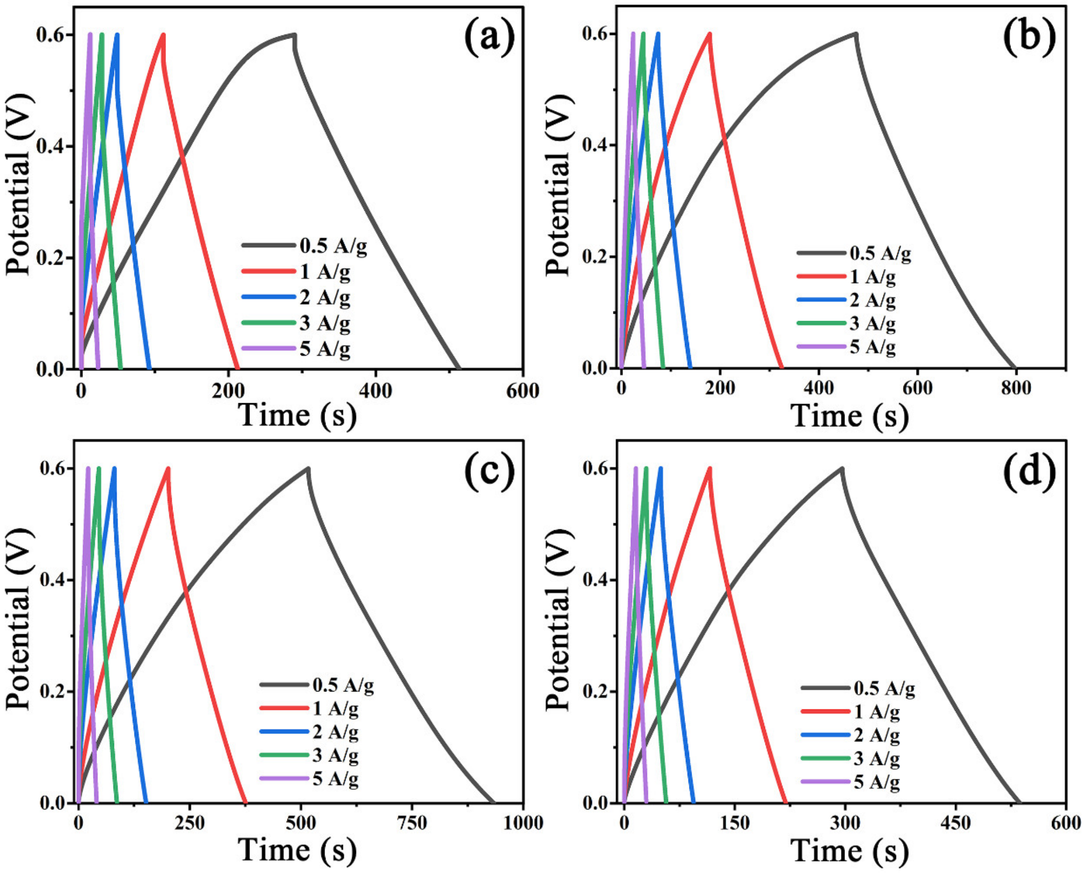

Figure S6 GCD curves at different current densities ranging from 0.5 to 5 A/g of (a) VO_x, (b) V@P-1, (b) V@P-3, and (b) V@P-5 electrode.

Table S1 The content of C, N, O, V elements for V@P-3 composite by EDS analysis.

Element	Weight/%	Atomic/%	Net error/%
C K	33.56	51.11	0.50
N K	16.31	21.30	2.34
O K	12.21	13.97	2.64
V K	37.92	13.62	0.78

Table S2 Impedance values of the VO_x and V@P-3 according to the equivalent circuit.

Samples	Parameters			
	R_0 (Ω)	R_{ct} (Ω)	C_p (F)	Z_W (Ω)
VO_x	3.16	0.48	0.98	0.39
V@P-3	3.01	0.12	1.12	0.44

Table S3 Comparison of areal power density and energy density for VO_x@PANI SSC and other reported vanadium oxide-based supercapacitors.

Supercapacitor	Energy density	Power density	Refs.
α-V_2O_5 SSC	0.48 μWh/cm^2	0.11 mW/cm^2	[1]
V_2O_5/PDOTE SSC	11.00 μWh/cm^2	0.19 mW/cm^2	[2]
V_2O_5@PDOTE/graphene SSC	0.18 μWh/cm^2	0.01 mW/cm^2	[3]
$V_2O_5 \cdot H_2O$/graphene SSC	1.13 μWh/cm^2	0.01 mW cm^2	[4]
MnO_2/V_2O_5@MWCNT SSC	6.58 μWh/cm^2	0.20 mW/cm^2	[5]
VO_x@PANI SSC	115.17 μWh/cm^2	0.39 mW/cm^2	this work

Reference

1. Adewinbi, S.A.; Busari, R.A.; Animasahun, L.O.; Omotoso, E.; Taleatu, B.A. Effective pseudocapacitive performance of binder free transparent α-V_2O_5 thin film electrode: Electrochemical and some surface probing. *Physica B: Condensed Matter*, **2021**, 621: 413260.
2. QI, R.J.; Nie, J.H.; Liu, M.Y.; Lu, X.M. Stretchable V_2O_5/PEDOT Supercapacitors: A Modular Fabrication Process and Charging with Triboelectric Nanogenerators. *Nanoscale*, **2018**, 10: 7719-7725.
3. Wang, L.B.; Shu, T.; Guo, S.T.; Lu, Y.; Li, M.X.; Nzabahimana, J.; Hu, X.L. Fabricating strongly coupled V_2O_5@PEDOT nanobelts/graphene hybrid films with high areal capacitance and facile transferability for transparent solid-state supercapacitors. *Energy Storage Materials*, **2020**, 27: 150-158.
4. Bao, J.; Zhang, X.D, Bai, L.F.; Bai, W.C.; Zhou, M.; Xie, J.F.; Guan, M.L.; Zhou, J.F.; Xie, Y. All-solid-state flexible thin-film supercapacitors with high electrochemical performance based on a two-dimensional $V_2O_5 \cdot H_2O$/graphene composite. *Journal of Materials Chemistry A*, **2014**, 2(28): 10876-10881.
5. Park, H.; Song C.; Jin, S.W.; Lee, H.; Keum, K.; Lee, Y.H.; Lee, G.; Jeong, Y.R.; Ha, J.S. High performance flexible micro-supercapacitor for powering a vertically integrated skin-attachable strain sensor on a bio-inspired adhesive. *Nano Energy*, **2021**, 83: 105837.
